# Wenyang Jieyu Decoction Alleviates Depressive Behavior in the Rat Model of Depression via Regulation of the Intestinal Microbiota

**DOI:** 10.1155/2020/3290450

**Published:** 2020-07-23

**Authors:** Zhenyu Feng, Xiaojuan Ma, Shuang Meng, Hongjuan Wang, Xiaorong Zhou, Min Shi, Jie Zhao

**Affiliations:** ^1^Central Laboratory, Shanxi Hospital of Integrated Traditional and Western Medicine, Taiyuan City 030013, Shanxi Province, China; ^2^Graduate School, Shanxi University of Chinese Medicine, Taiyuan City 030619, Shanxi Province, China; ^3^Department of Traditional Chinese Medicine, Shanxi Hospital of Integrated Traditional and Western Medicine, Taiyuan City 030013, Shanxi Province, China

## Abstract

**Background:**

Intestinal microbiota plays an important role in the occurrence and treatment of depression. This study investigated whether Wenyang Jieyu decoction (WYJYD) alleviates depressive behavior in the rat model via regulation of the intestinal microbiota.

**Methods:**

Rat model of depression was established by stress stimulus. SD male rats were randomly allocated into normal control, model, model + low-dose WYJYD (1.89 g/kg/d), model + medium-dose WYJYD (3.08 g/kg/d), model + high-dose WYJYD (7.56 g/kg/d), and model + fluoxetine (3.33 mg/kg/d) groups. Behavioral changes were observed using forced swim test. Histopathological changes in hippocampal tissue were examined by HE staining. Indicators in serum were detected by ELISA. Indicators in hippocampal tissue were detected by qPCR and western blot. Microbiota distribution in feces was detected using high-throughput 16S rRNA gene sequencing.

**Results:**

Compared with the model group, the immobility time in WYJYD and fluoxetine groups was significantly decreased (*P* < 0.05), and the cell structure was significantly improved. Compared with the model group, the 5-hydroxytryptamine (5-HT) and norepinephrine (NE) levels in medium- and high-dose WYJYD groups and the brain-derived neurotrophic factor (BDNF) level in the high-dose WYJYD group were significantly increased (*P* < 0.05, all), and the fibroblast growth factor-2 (FGF2), forkhead box protein G1 (FOXG1), and phospho-protein kinase B/protein kinase B (p-AKT/AKT) expressions were increased with WYJYD treatments. The Chao1 and ACE indices in high-dose WYJYD and the Simpson and Shannon indices in medium-dose WYJYD were significantly different than the model group. The similarity of the intestinal microbial community of each group after WYJYD treatment tended to be closer to the control group. Compared with the model group, as the dosage of WYJYD increased, the abundance of genera *Coprococcus*, *Lachnospira*, and *rc4-4* was significantly increased, while the abundance of genera *Desulfovibrio*, *Burkholderia*, and *Enterococcus* was significantly decreased.

**Conclusion:**

WYJYD may alleviate the depressive behavior of the rat model by regulating the intestinal microbiota and neurotransmitters.

## 1. Introduction

Depression is a common type of mental illness, with a high clinical incidence. Its main manifestations include depressed, sadness, frustration, despair, and other emotional symptoms [1]. Patients are often accompanied by various degrees of sleep disorders and social fear. In severe cases, there is a higher suicidal tendency, which seriously affects daily life and career development [2]. Survey shows that depression has become one of the diseases with higher incidence, and the number of patients is increasing yearly. According to the latest report of the WHO, by 2020, depression will become the second largest category of diseases that causes human death and disability [3, 4].

At present, there are many ways to treat depression clinically, mainly including drug therapy, psychological therapy, and physical therapy. Commonly used antidepressants are 5-hydroxytryptamine (serotonin, 5-HT), sertraline, and fluoxetine [5, 6], as well as newly developed steroid drugs with nervous system targeting and hormones [7]. The onset of psychotherapy is a gradual and cumulative effect process, which is slower than the onset of drugs. Generally, drug therapy is supplemented by psychotherapy [8]. Physical therapy mainly includes electroconvulsive therapy (ECT), vagus nerve stimulation (VNS), and transcranial magnetic stimulation (TMS). They all have certain effects and are usually used mainly as supplementary therapies [9]. Due to its convenience and rapid effect, drug treatment has become the most commonly used method in clinical practice. However, the long-term effect of antidepressant medication has not been satisfactory, easy to produce side effects, and the disease is easy to recur.

According to the theory of traditional Chinese medicine (TCM), depression is caused by excessive emotion or constrain for a long time that cannot be vented. The pathogenesis of depression is related to the five internal organs, and the disease location mostly involves the Liver, Heart, Spleen, Lung, and Kidney. Based on the fact that the Liver is in charge of catharsis and can regulate emotional activities, it is believed that depression is caused by stagnation of Liver Qi, and the treatment is emphasized on soothing the Liver and regulating Qi [10].

Wenyang Jieyu decoction (WYJYD) comprises *Ramulus Cinnamomi*, *Radix Aconiti Lateralis Preparata*, *Radix Glycyrrhizae Preparata*, *Zingiber officinale* Roscoe, *Fructus mume*, and *Ziziphus jujuba* Mill. WYJYD has the effects of invigorating the Kidney and warming the Spleen, nourishing Qi and regenerating body fluid, relieving the Liver, and regulating Qi. It has good therapeutic effect on depression, but due to the diverse active ingredients of Chinese medicine, the mechanism is unclear [11]. It has been shown to have good clinical efficacy, and it is safe in treating depression [12–14].

The main component and major active component in *Ramulus Cinnamomi* is cinnamaldehyde, which is known for its sedative, antioxidant, and antineuroinflammation activity [15–19]. Several other active components in its extract have also been reported to have neuroprotective effect [20–22]. Combination of *Ramulus Cinnamomi* extracts with *Lactobacillus* has been shown to alter intestinal microbiota, alleviate high-fat diet-induced obesity, and mainly increase *α*-diversity [23], which may produce beneficial effects on depression influenced by obesity [24]. Gyejibokryeong-hwan, a traditional medicine which comprises herbs including *Ramulus Cinnamomi*, can ameliorate depressive-like behavior in mice with reserpine-induced depression [25].

Previous study showed that *Radix Aconiti Lateralis Preparata* can suppress proinflammatory factors IL-9, IL-13, and PGE-2 [26]. Wu Mei Wan which consists of herbal materials including *Ramulus Cinnamomi, Radix Aconiti Lateralis Preparata,* and *Fructus mume*, can regulate the intestinal microbiological structure, balance the “tumor-promoting bacteria” and “tumor-suppressing bacteria,” and attenuate colorectal cancer [27]. *Radix Aconiti Lateralis Preparata* and its components have been frequently prescribed as treatment for depression in TCM [28, 29], and its polysaccharide has been reported to have antidepressant-like effect [29].

*Glycyrrhiza* sp. have long been used worldwide as a herbal medicine. Clinical and experimental studies suggested that they have several useful pharmacological properties such as anti-inflammatory, antiviral, antimicrobial, antioxidative, and anticancer activities, immunomodulatory effects [30], and neuroprotective effects [31]. Previous study showed that Jianpi mixture which consists of herbs including *Radix Glycyrrhizae Preparata* can improve the species diversity of intestinal microbiota in mice with diarrhea irritable bowel syndrome [32]. Danzhi Xiaoyao powder which comprises herbal materials including *Radix Glycyrrhizae Preparata* has been found to have a good antidepressant effect [33].

*Zingiber officinale* Roscoe has been shown to have antioxidant, anticancer, anti-inflammatory, and antiapoptotic effects [34]. Previous study revealed that its supplementation can modulate the intestinal microbiota and has therapeutic effect on obesity in mice [35]. Its extract has been shown to have significant antidepressant activity in the forced swim test (FST) model in mice [36].

*Fructus mume* has been used as a traditional treatment for ulcer and digestive problems. It has been reported to be effective in the rat model of colitis [37], improved cognitive impairment in mice [38, 39], has anti-inflammatory effects [38, 39] and antidepression properties [40], and is inhibitory to proinflammatory mediators [41]. *Fructus mume* extracts have been a widely accepted treatment for various kinds of diarrhea [42]. It can alleviate diarrhea in breast cancer patients [40]. Patients with diarrhea often have psychiatric comorbidities [43], and alterations in the intestinal microbiota have been regarded to be associated with depression [44].

*Ziziphus jujuba* Mill. has been shown to have various biological effects, including immunomodulatory, antioxidant, antitumor, and gastrointestinal-protective effects [45]. Previous study showed that it can modify the intestinal microbiota of the mouse colon tumor model [46] and restore the intestinal microbiota profile altered by azoxymethane (AOM)/dextran sodium sulfate (DSS) [47]. It is also a potential candidate for treatment of neurological diseases [48].

Recent studies have shown that intestinal microbiota plays an important role in the occurrence and treatment of depression and has become a new target for the prevention and treatment of depression [49]. Evidence showed microbiota composition changes as a response to a stressful situation, and the gut-brain axis influences stress [50]. A better understanding of the mechanism of the underlying stress modulation through the microbiota is important for the design of novel therapeutics for stress-related disorders such as depression.

Therefore, this study is designed to investigate the effect of stress on the intestinal microbiota and depression and the influence of WYJYD treatment on the rat model of depression. We hypothesized that WYJYD exerts its antidepressant effect by regulating the diversity, structure, and function of the interconnecting microbiota and the abundance of specific taxa. The hypothesis was verified by examining the indicators in rats and analyzing the fecal microbiota diversity. The aim of the present work is to explore whether WYJYD alleviates depression in the rat model via regulation of the intestinal microbiota, and the findings of this study could help to provide theoretical and experimental evidence for the treatment of depression.

### 1.1. Ethnopharmacological Relevance

WYJYD comprises medicinal herbs which have been used as TCM for thousands of years and chronicled in “Yi Fa Yuan Tong” [51], “Compendium of Materia Medica (Ben Cao Gang Mu) [52], and “Shen Nong Ben Cao Jing” [53] and is effective in treating depression. This study is performed to investigate its underlying mechanism and explore the reason for its effect. Future research will attempt to improve its role in treatment.

## 2. Materials and Methods

### 2.1. Ethical Approval

This study was approved by the Animal Use and Care Committee of Shanxi Hospital of Integrated Traditional and Western Medicine. All animal studies were conducted in accordance with the principles and procedures outlined in the National Institutes of Health (NIH) Guide for the Care and Use of Laboratory Animals (revised 2011).

### 2.2. Experimental Animals

Eight weeks old, adult male Sprague Dawley (SD) healthy rats, weighed 220 ± 10 g were purchased from Hunan SJA Laboratory Animal Co., Ltd, Hunan, P.R. China (license number: SCXK (Xiang) 2016-0002). All rats were kept in a specific-pathogen-free (SPF) animal room, maintained at constant temperature and humidity, under a standard 12 h light/dark cycle, and supplied with aseptic nutritional pellet feed and sterile water *ad libitum*.

### 2.3. Drugs, Main Reagents, and Instruments

The drugs, main reagents, and instruments used in this study are as follows: WYJYD powder (self-made); fluoxetine hydrochloride dispersible tablets (Eli Lilly and Co., Alcobendas, Madrid, Spain); Invitrogen TRIzon reagent, ultrapure RNA extraction kit, HiFiScript cDNA first-strand synthesis kit, UltraSYBR mixture, bicinchoninic acid (BCA) protein assay kit (CWBIO, Beijing, P.R. China); radioimmunoprecipitation assay (RIPA) cell lysis buffer, nonfat-dried milk (for blocking) (Applygen Technologies Inc., Beijing, P.R. China); bovine serum albumin (BSA), Scott bluing buffer (Solarbio Life Sciences, Beijing, P.R. China); mouse monoclonal antiglyceraldehyde 3-phosphate dehydrogenase (GAPDH) (1 : 2000); goat anti-mouse IgG (*H* + *L*) horseradish peroxidase (HRP) conjugate (1 : 5000), goat anti-rabbit IgG (*H* + *L*) HRP conjugate (1 : 5000) (Zsbio Commerce Store, Beijing, P.R. China); rabbit anti-protein kinase B (PKB, also known as AKT) (AKT1) (1 : 1000), rabbit anti-phospho- (p-) AKT (1 : 1000) (Bioss Antibodies Inc., Woburn, MA, USA); rabbit anti-forkhead box G1 (FOXG1) (1 : 1000) (ABCAM, Cambridge, MA, USA); rabbit anti-fibroblast growth factor-2 (FGF2) (1 : 1000) (Affinity Biosciences, Cincinnati, OH, USA); hematoxylin, eosin (Boster Biological Technology, Pleasanton, CA, USA); superclean advanced adhesive sealant (BaSO Diagnostics Inc., Zhuhai, Guangdong, P.R. China); rat brain-derived neurotrophic factor (BDNF) enzyme-linked immunosorbent assay (ELISA) kit, rat 5-HT ELISA kit, rat norepinephrine (NE) ELISA kit (Jiangsu Meimian Industrial Co., Ltd, Zhangjiagang, Jiangsu, P.R. China); CFX Connect^™^ fluorescent real-time polymerase chain reaction (PCR), ultrahigh sensitivity chemiluminescence imaging system (ChemiDoc^™^ XRS+) (Bio-Rad Laboratories, Shanghai, P.R. China); freezing high-speed centrifuges (TGL-16G) (Changzhou ZOJE Experimental Instrument Manufacturing Co., Ltd., Jiangsu, P.R. China); UV-visible spectrophotometer (UV-1600PC, Shanghai Mapada Instruments Co., Ltd., Shanghai, P.R. China); vertical protein electrophoresis system (DYY-6C, Beijing Liuyi Instrument Factory, Beijing, P.R. China); multifunctional microplate reader (S/N502000011, TECAN, Männedorf, Zürich, Switzerland); microscope (CX41, Olympus Co., Shinjuku, Tokyo, Japan); and slicer (BQ-318D, Borner, Niederkail, Rhineland-Palatinate, Germany).

### 2.4. Preparation of WYJYD TCM Powder

Composition of Wenyang Jieyu decoction: *Ramulus Cinnamomi*, *Radix Aconiti Lateralis Preparata* 15 g each, *Radix Glycyrrhizae Preparata* 10 g, *Zingiber officinale* Roscoe and *Fructus mume* 30 g each, 5 *Ziziphus jujuba* Mill. The above medicinal herbs were weighed in proportion. First, *Radix Aconiti Lateralis Preparata* was taken, added 12 times amount of water, and boiled for 1 h. Then, the remaining herbal medicines were taken, added 12 times amount of water, decocted, and extracted 3 times, for 1 h each. The decoction obtained from both procedures was combined and filtered. The filtrate was concentrated under reduced pressure to an extract of relative density 1.2 (80°C), dried, crushed into fine powder, and reserved for use.

### 2.5. Experimental Grouping and Animal Modeling

The experiment was randomly allocated into 6 groups: normal control group (control), depression model group (model), model + low-dose WYJYD group (model + low dose), model + medium-dose WYJYD group (model + medium dose), model + high-dose WYJYD group (model + high dose), and model + fluoxetine group (model + fluoxetine) (positive control) (*n* = 6 in each group).

### 2.6. Establishment of the Rat Model of Depression

The experimental rats were adaptively fed for one week. Then, each rat was raised in a single cage. The rat model was established by human interference with various mild stimulation methods: tail clipping (1 min), noise (1 h), flash (1 h), ice water swim (4°C, 5 min), wet padding (100 mL, 24 h), tilting of the rat cage (30°, 24 h), day and night reversal, and food and water banning. Random stimulation methods were arranged daily between 9:00 and 16:00 for 28 days. The rats were given 1 stimulus per day, and each stimulus would appear at least 3 times. The same stimulus could not appear continuously to prevent the rats from adapting to the stimulus.

At the same time as the rat model of depression was established, the rats were given intragastric administration according to the specified group:

1. Normal control group (control): the rats were not given any treatment
2. Depression model group (model): the rats were given 2 ml/kg/d physiological saline
3. Model + low-dose WYJYD group (model + low dose): the rats were given 1.89 g/kg/d WYJYD powder dissolved in water
4. Model + medium-dose WYJYD group (model + medium dose): the rats were given 3.08 g/kg/d WYJYD powder dissolved in water
5. Model + high-dose WYJYD group (model + high dose): the rats were given 7.56 g/kg/d WYJYD powder dissolved in water
6. Model + fluoxetine group (model + fluoxetine): the rats were given 3.33 mg/kg/d fluoxetine dissolved in water

### 2.7. Behavioral Test-FST

The FST [54] is a rodent behavioral test used to evaluate “depressive-like” states and behavioral despair. It was developed to test antidepressant efficacy of new compounds. It is based on the assumption that when placing an animal in a container filled with water, it will first make efforts to escape but eventually will exhibit immobility that may be considered to reflect a measure of behavioral despair. It involves exposure of animals to stress, which was shown to have a role in the tendency for major depression.

The rats were forced swimming for two days. On the first day, a cylindrical container with a diameter of 20 cm and a height of 40 cm was used. The water depth was that the rat would not be able to touch the bottom of the container. The water temperature was 23°C–25°C. On the first day of the experiment, the rats were placed in water for 15 min for forced swimming training. After 24 h of training, the rats were forced to swim for 6 min. The swimming behavior of the rats and their immobility time (the time when the rat had no other behavior except to move upward to avoid submersion in water) were recorded.

### 2.8. Animal Sampling

After the behavioral test, the rats in each group were anesthetized with intraperitoneal (IP) injection of 45 mg/kg sodium pentobarbital and sacrificed by cervical dislocation. The rat's skull was opened, and the entire brain was gently removed with small forceps. The extra parts were removed, and the brain was divided into 2 along the midline with a surgical blade, in which half was fixed with hematoxylin-eosin (HE) staining for detection, while for the other half, the hippocampus was separated and divided into three and cryopreserved for ELISA, quantitative polymerase chain reaction (qPCR), and western blot (WB) detection. The colon contents of the rats were taken, put into sterile cryopreservation tubes, and kept for 16S rRNA gene sequencing of microbial diversity.

### 2.9. HE Staining

The tissue was removed and rinsed with water for several hours and dehydrated in 70%, 80%, and 90% ethanol solution. Then, it was placed in the same amount of mixed pure alcohol and xylene for 15 min and in xylene I and xylene II for 15 min each until clear followed by mixed solution of xylene and paraffin in equal amount for 15 min and in paraffin I and II for 50–60 min each. The specimen was paraffin-embedded and sliced. The paraffin sections were baked, dewaxed, and hydrated. The sections were placed in distilled water, and hematoxylin aqueous solution was added for 3 min. This was followed by hydrochloride ethanol differentiation for 15 s, slightly washed with water, and placed in bluing buffer for 15 s. The specimen was then washed with running water and eosin-stained for 3 min, washed with running water, cleared, mounted, and examined under a microscope. The successful establishment of the rat model of depression was determined. Histopathological changes in the treatment groups with WYJYD or fluoxetine were also examined.

### 2.10. ELISA Test

All reagents and kit components were restored to room temperature (RT). The standard wells and sample wells were set, and 50 *μ*L of standards at different concentrations was added to the standard wells. The blank (normal control) wells were set, awaiting sample testing. Then, 40 *μ*L of sample diluent was added to the enzyme-coated plate followed by 10 *μ*L of the sample. Next, 100 *μ*L of the enzyme-labeled reagent was added to each well, except for the blank wells. The plate was sealed with a sealing film and incubated at 37°C for 60 min. Then, the sealing film was carefully removed, and the liquid was discarded, dried, washed, and patted dry. Each well was added with 50 *μ*L of developer A followed by 50 *μ*L of developer B and mixed by gentle shaking. Color was developed at 37°C in the dark for 15 min. Then, 50 *μ*L of stop solution was added in each well to terminate the reaction. The optical density (OD) value of each well was measured at 450 nm.

### 2.11. Real-Time qPCR

The tissue samples were ground into powder in a mortar under liquid nitrogen. A corresponding amount of the TRIzon reagent was added, and then collected with a pipette in a centrifuge tube prepared in advance. Then, chloroform was added, vigorously shaken for 15 s, and left at RT for 5 min. The samples were then centrifuged at 13,400 rpm for 20 min at 4°C. The aqueous phase was collected, and an equal volume of precooled 70% ethanol was added and mixed by inversion. Then, this was loaded onto the RM adsorption column of the collection tube and centrifuged at 12,000 rpm for 30 s at 4°C. The effluent was discarded and left to dry for 5 min at RT. The obtained RNA was stored at −80°C to prevent degradation. The primer information is shown in Table 1. The primers were synthesized by Sangon Biotech (Shanghai) Co., Ltd., Shanghai, P.R. China.

The operating system for real-time qPCR and reaction procedure are shown in Tables 2 and 3.

### 2.12. WB

The sodium dodecyl sulfate-polyacrylamide gel electrophoresis (SDS-PAGE) separating gel and stacking gel were configured as required and added to the prepared gel plate slowly until 2/3 of the plate. Absolute ethanol was added to press the gel. After the separating gel was solidified, the stacking gel was added, and the tooth comb was inserted. After the stacking gel had been completely solidified, 10x electrophoresis buffer was diluted, the frame was added into the electrophoresis solution, and the tooth comb was removed. The protein sample and marker were added. Electrophoresis was started, and 60 V and 80 V were applied to compact and separate the protein, respectively. When the band was halfway through the gel plate, transfer solution was prepared and precooled. The polyvinylidene fluoride (PVDF) membrane was cut according to the expected position of the band, and the membrane was activated by incubation in methanol for 15 s. The appropriate size of the gel including the target band was cut. The “sandwich” (sponge-filter paper-gel-membrane-filter paper-sponge) was prepared, and membrane transfer was conducted. Overnight blocking was performed using 5% BSA blocking solution. The PVDF membrane was incubated with primary antibodies at 4°C overnight. Then, the membrane was washed for 10 min (3x) and incubated with the secondary antibody at RT for 1 h. The membrane was then washed for 10 min (3x). Drops of enhanced chemiluminescent (ECL) solution was added to the membrane and exposed under the gel imaging system.

### 2.13. Microbiota Diversity Analysis

After filtering the intestinal contents of the rats in each group, total DNA was extracted and quantified by using the spectrophotometer. Generally, target sequences such as microbial ribosomal RNAs that could reflect the composition and diversity of the microbiota were used as targets, and the corresponding primers were designed based on the conserved regions in the sequence for PCR amplification. The PCR-amplified products were detected by 2% agarose gel electrophoresis, and the target fragments were cut and recovered. The recovered PCR-amplified products were quantified by fluorescence and subjected to high-throughput sequencing. Using Quantitative Insights Into Microbial Ecology (QIIME) software, the UCLUST sequence comparison tool was invoked. The sequences obtained by high-throughput sequencing were merged, and the operational taxonomic unit (OTU) was divided according to 97% sequence similarity. The sequence with the highest abundance in each OTU was selected as the representative sequence of the OTU, and the Ribosomal Database Project (RDP) database was compared to obtain the classification and identification results.

### 2.14. Statistical Analysis

SPSS 19 (SPSS Inc., Chicago, Illinois, USA) was used to statistically analyze the obtained data. The measurement data were expressed as mean ± standard deviation $\left(\bar{x}\pm s\right)$. T-test was used for comparison between two groups, and one-way analysis of variance (ANOVA) was used for comparison between multiple groups. *P* < 0.05 was considered as statistically significant.

## 3. Results

### 3.1. Behavioral Analysis of Rats Showed Decrease in Immobility Time with WYJYD Treatment

**Figure 1:**
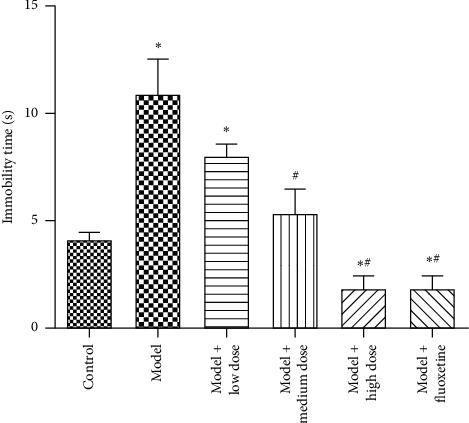
Immobility time analysis in FST of rats. Note: compared with the control group, ^*∗*^*P* < 0.05; compared with the model group, ^#^*P* < 0.05. The data are presented as mean ± standard deviation. FST: forced swim test.

The immobility time in the model group was significantly longer than that in the normal control group (*P* < 0.05) and was the longest among the groups (*P* < 0.05). The immobility time of the WYJYD groups decreased significantly with the increase of dose, and the fluoxetine group was also decreased significantly compared with the model group (*P* < 0.05) (Figure 1).

### 3.2. HE Staining of Hippocampal Tissue Showed Improvement with WYJYD Treatment

**Figure 2:**
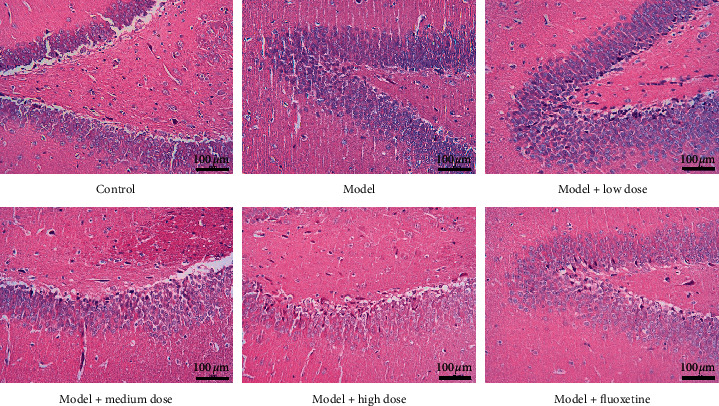
HE staining of the hippocampus in each group of rats. HE: hematoxylin-eosin.

HE staining of the hippocampal tissue was performed in each group of rats. In the normal control group, the pyramidal cells were arranged neatly, with dense layers, regular, and clear structure. The model group demonstrated loosely arranged pyramidal cells, with missing cells and a fuzzy structure. This indicates that the rat model of depression was successfully established. The WYJYD and fluoxetine groups showed a certain degree of damage, and the cell arrangement was fuzzy but significantly improved compared with the model group (Figure 2).

### 3.3. ELISA Test Showed Improvement Changes in 5-HT, BDNF, and NE Levels with WYJYD Treatment

**Figure 3:**
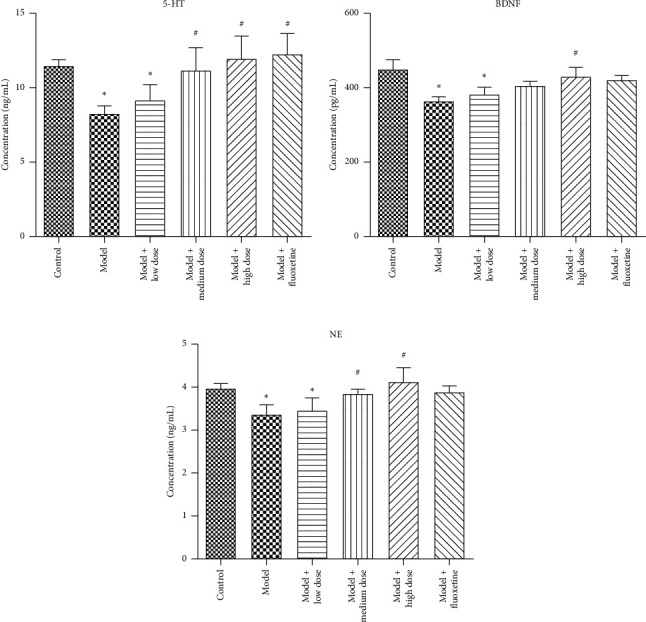
ELISA detection of 5-HT, BDNF, and NE levels in serum. Note: compared with the control group, ^*∗*^*P* < 0.05; compared with the model group, ^#^*P* < 0.05. All data are presented as mean ± standard deviation. ELISA: enzyme-linked immunosorbent assay, 5-HT: 5-hydroxytryptamine, BDNF: brain-derived neurotrophic factor, and NE: norepinephrine.

Compared with the normal control group, the 5-HT, BDNF, and NE levels in the model group were significantly decreased (*P* < 0.05, all). After treatment with WYJYD, the 5-HT and NE levels in the medium- and high-dose groups and the BDNF level in the high-dose group were significantly increased compared with the model group (*P* < 0.05, all), and overall was better than the fluoxetine group (Figure 3).

### 3.4. qPCR and WB Showed Increase in FGF2, FOXG1, and p-AKT/AKT Expressions with WYJYD Treatment

**Figure 4:**
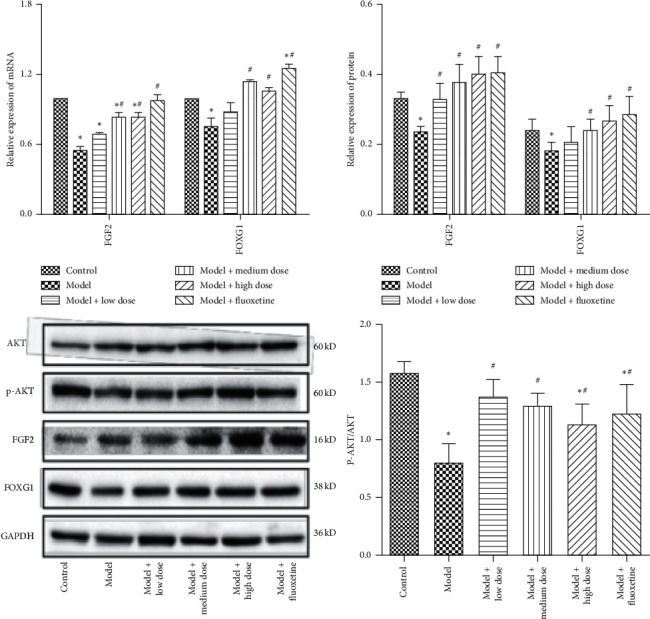
qPCR and WB detection of FGF2, FOXG1, and p-AKT/AKT expressions. Note: compared with the control group, ^*∗*^*P* < 0.05; compared with the model group, ^#^*P* < 0.05. All data are presented as mean ± standard deviation. qPCR: quantitative polymerase chain reaction, WB: western blot, FGF2: fibroblast growth factor-2, FOXG1: forkhead box protein G1, and p-AKT/AKT: phospho-protein kinase B/protein kinase B.

Compared with the normal control group, the FGF2, FOXG1, and p-AKT/AKT expressions in the model group were significantly decreased (*P* < 0.05, all). The expressions were increased after treatment with WYJYD or fluoxetine (Figure 4).

### 3.5. Microbiota Diversity Analysis Showed Changes in *α*-Diversity Indices and Intestinal Microbial Community after WYJYD Treatment

**Figure 5:**
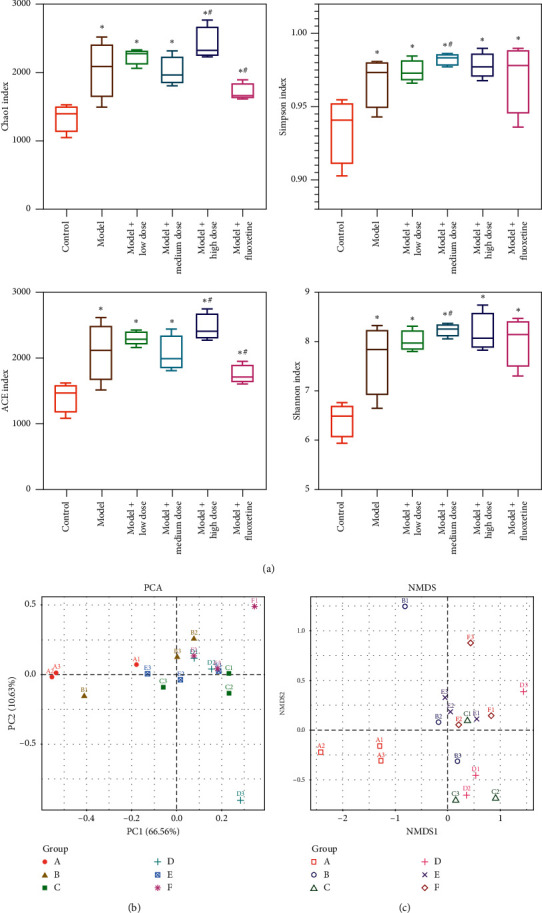
Microbiota diversity analysis of rat feces. (a) *α*-Diversity index analysis, (b) PCA analysis of *β*-diversity, and (c) NMDS based on UniFrac distance. (A) Control group, (B) model group, (C) model + low-dose WYJYD group, (D) model + medium-dose WYJYD group, (E) model + high-dose WYJYD group, and (F) model + fluoxetine group. Note: compared with the control group, ^*∗*^*P* < 0.05; compared with the model group, ^#^*P* < 0.05. PCA: principal component analysis; NMDS: nonmetric multidimensional scaling.

Figure 5(a) shows the *α*-diversity indices of the 6 groups. It can be seen that Chao1, Simpson, ACE, and Shannon, 4 diversity indices of the model group were significantly different than the control, indicating significant changes in the intestinal microbiota after establishment of the rat model of depression compared with normal rats. After treatment with WYJYD and fluoxetine, the Chao1 and ACE indices of the high-dose WYJYD group and the Simpson and Shannon indices of the medium-dose WYJYD group were significantly different compared with the model group. The changes in the fluoxetine (positive control) group were most obvious.  Figure 5(b) shows the principal component analysis (PCA) of *β*-diversity.  Figure 5(c) demonstrates nonmetric multidimensional scaling (NMDS) based on UniFrac distance. It showed that, after treatment with WYJYD, the similarity of the intestinal microbial community of each group tended to be closer to the normal group, indicating improvement in the intestinal microbiota.

### 3.6. Relative Abundance Analysis and Genera Level Classification Composition Showed Changes with WYJYD Treatment

**Figure 6:**
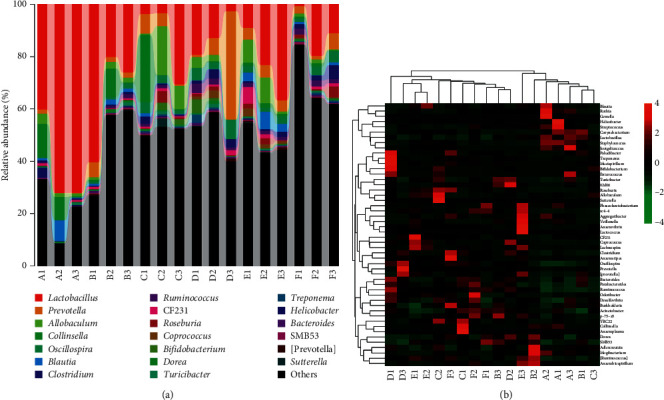
Differential analysis of genera level in rat feces. (a) Genera level classification composition and relative abundance. (b) Heat map of genera level community composition combined with cluster analysis. (A) Control group, (B) model group, (C) model + low-dose WYJYD group, (D) model + medium-dose WYJYD group, (E) model + high-dose WYJYD group, and (F) model + fluoxetine group.

Compared with the normal control group, the genera in the model group with significantly reduced abundance were *Rothia*, *Streptococcus*, *Staphylococcus*, and *Jeotgalicoccus*, while the genera with significantly increased abundance were *Dorea*, *Parabacteroides*, *Oscillospira*, and *Prevotella*. After administration of WYJYD, the genera abundance showed significant changes. With the increase of dosage, the genera with significantly increased abundance were *Coprococcus*, *Lachnospira*, and *rc4-4*, while the genera with significantly decreased abundance were *Desulfovibrio*, *Burkholderia*, and *Enterococcus* (Figure 6).

## 4. Discussion

Studies have shown that antidepressants have an effective rate of about 60% to 80% and a cure rate of only 30%. Although the effect is rapid, they can only cure the symptoms but not the root cause, and the disease is easy to recur. Over time, patients become dependent on the drugs [55, 56]. TCM treatment of depression emphasizes on holistic and overall views that treat both symptoms and root cause of the disease. Although the effect is slow, it is mild, and the patient will not develop drug dependence; thus, the condition is not easy to recur.

WYJYD was used to treat depression in the rat model. This study found that the behavioral performance of the treated rats tended to be normal, and the immobility time of the high-dose WYJYD group and the fluoxetine (positive control) group in FST was even shorter than that of the normal group. The results of HE staining of the hippocampal tissue also showed a certain improvement after administration of WYJYD, and the cell arrangement tended to be neat. ELISA detection of 5-HT, BDNF, and NE in serum also indicates that the levels in the WYJYD groups are close to the normal group, suggesting that WYJYD had a certain therapeutic effect. FGF2 plays an important role in promoting the generation, survival, and repair of neurons. It is mainly involved in the growth and regeneration of neurons and is very closely related with depression. At present, it has become a hotspot in the studies on neurodegenerative diseases [57]. FOXG1 plays an important role in the differentiation and tangential migration of interneurons. When the FOXG1 gene is destroyed, the axons of the interneurons become shorter, the dendrites are reduced, and they cannot migrate to the cerebral cortex, which can cause excitation and inhibition imbalance of the cortical circuit [58]. Studies have shown that phosphorylation of AKT is related to the relief of depression. It initiates the phosphatidylinositol 3-kinase (PI3K)/Akt signal transduction pathway, regulates the survival and proliferation of neurons, forms the signal transmission chain that promotes neuron growth, inhibits apoptosis during stress, and repairs damaged brain tissue [59]. According to the results of this study, administration of WYJYD promotes the expressions of FGF2 and FOXG1, and AKT phosphorylation in the hippocampus of the rat model of depression and alleviates depression symptoms.

Intestinal dysbiosis is closely related to the occurrence of various diseases, such as asthma, obesity, diabetes, and particularly mental illness [60, 61]. In as early as 1987, questionnaire results showed that more than 60% of patients with intestinal diseases were associated with mental illness [62]. Clinical research showed that the intestinal microbiota of patients with depression is abnormal, which is mainly manifested by abnormal *α*-diversity indices, reduced abundance of Spirillaceae, and increased abundance in the Firmicutes and Bacteroidetes ratio and the Bacteroidales and *Fusobacterium* ratio [63]. According to the results of this study, the genera with significantly reduced abundance in the intestinal microbiota in the rat model of depression were *Rothia*, *Streptococcus*, *Staphylococcus*, and *Jeotgalicoccus*, and the genera with significantly increased abundance were *Dorea*, *Parabacteroides*, *Oscillospira*, and *Prevotella*. It can be explained that the intestinal microbiota in depressive rats had changed significantly compared with normal rats, and after treatment with WYJYD, the microbiota was changed significantly compared with the model rats. With the increase in dosage, the genera with significantly increased abundance were *Coprococcus*, *Lachnospira*, and *rc4-4*, and the genera with significantly decreased abundance were *Desulfovibrio*, *Burkholderia*, and *Enterococcus*. It shows that the intestinal microbiota affects the development of the cerebral nervous system to a certain extent, while damage to the cerebral nervous system also affects the balance of the intestinal microbiota. Study by Collins et al. explained that the intestinal microbiota and its products can affect the brain function through blood circulation and other ways [64] and may as well stimulate intestinal endocrine cells to secrete 5-HT, BDNF, NE, and other substances to affect the brain function, thereby reducing stress and mood, and alleviating depression.

The relationship between the intestinal microbiota and the brain leads to development of psychobiotic-based therapeutic strategies [65]. Recently, there have been an increasing number of studies investigating the interaction between the intestinal microbiota and herbal medicines. Previous study found that interactions between the intestinal microbiota and herbal medicines can be attributed to the absorbable active small molecules, and changed intestinal microbiota and its secretion. Herbal medicine can regulate the composition of the intestinal microbiota, and at present, technology such as 16S rRNA sequencing can detect the levels of related microbiota but unable to evaluate the specific role of the individual microbiota. By overcoming the technical limitations associated with intestinal microbiota detection and recognition, it would be possible to selectively regulate the intestinal microbiota levels through the use of drugs [66].

TCM has no drug dependence nor side effects, but it has the disadvantages of slow effect, complex components, and difficult to clarify the drug mechanism. Therefore, in its application, besides maintaining its benefits, it is necessary to further simplify the drug components, increase the content of active components, and accelerate the production of drug effects. It can also be used in combination with small molecule drugs, and combined treatment in depression can also produce unexpected results.

Further research in the future will include using modern techniques to extract the effective active components of WYJYD and explore its efficacy, further analyze the metabolites of the intestinal microbiota of rats after treatment to find out the main metabolites and investigate its specific mechanism, and combine the extracted active components with 5-HT or fluoxetine to treat depression and evaluate the effect.

## 5. Conclusions

Treatment with WYJYD improves the immobility time in FST, the levels of serum indicators (5-HT, BDNF, and NE), the expressions of hippocampal tissue indicators (FGF2, FOXG1, and p-AKT/AKT), the intestinal microbiota, *α*-diversity indices, and genera abundance in the rat model of depression. WYJYD can alleviate the depressive behavior in the rat model, and the effect may be related to the regulation of the intestinal microbiota and brain neurotransmitters, which provides a theoretical basis for WYJYD treatment in depression. Further studies are needed to validate the findings.

## Figures and Tables

**Table 1:** Primer information.

Primers	Primer sequence (5′-3′)	Primer length (bp)	Product length (bp)	Annealing temperature (°C)
AKT F	TACTGAGAACCGTGTCCTGC	20	289	58.0
AKT R	GTCCGTTATCTTGATGTGCC	20		
FOXG1 F	GAGGGCGACAAGAAGAACG	19	268	59.9
FOXG1 R	ACGGGTCCAGCATCCAGTAG	20		
FGF2 F	TCCATCAAGGGAGTGTGTGC	20	139	60.0
FGF2 R	TCCGTGACCGGTAAGTGTTG	20		
*β*-Actin F	ACGGTCAGGTCATCACTATC	20	90	56.6
*β*-Actin R	TGCCACAGGATTCCATACC	19		

**Table 2:** Operating system.

RNase-free dH_2_0	9.5 *μ*L
cDNA/DNA	1 *μ*L
Upstream primer	1 *μ*L
Downstream primer	1 *μ*L
2x ULtraSYBR mixture	12.5 *μ*L

**Table 3:** Reaction procedure (3 step method).

Steps	Temperature	Time
Pre-denaturation	95°C	10 min
Denaturing	95°C	10 s
Annealing	57.5°C	30 s
Extension	72°C	30 s
Cycle	40	

## Data Availability

All data generated or analyzed during this study are included in this published article.

## Additional Points

(i) Wenyang Jieyu decoction (WYJYD) improves the immobility time in forced swim test (FST) in the rat model of depression. (ii) WYJYD improves the levels of serum indicators (5-HT, BDNF, and NE) and the expressions of hippocampal tissue indicators (FGF2, FOXG1, and p-AKT/AKT) in the rat model of depression. (iii) WYJYD improves the intestinal microbiota, *α*-diversity indices, and genera abundance in the rat model of depression. (iv) WYJYD may alleviate the depressive behavior of the rat model via regulation of the intestinal microbiota and neurotransmitters.
